# Suramin Inhibits Hsp104 ATPase and Disaggregase Activity

**DOI:** 10.1371/journal.pone.0110115

**Published:** 2014-10-09

**Authors:** Mariana P. Torrente, Laura M. Castellano, James Shorter

**Affiliations:** 1 Department of Biochemistry and Biophysics, University of Pennsylvania, Philadelphia, Pennsylvania, United States of America; 2 Pharmacology Graduate Group, Perelman School of Medicine, University of Pennsylvania, Philadelphia, Pennsylvania, United States of America; National Center for Geriatrics and Gerontology, Japan

## Abstract

Hsp104 is a hexameric AAA+ protein that utilizes energy from ATP hydrolysis to dissolve disordered protein aggregates as well as amyloid fibers. Interestingly, Hsp104 orthologues are found in all kingdoms of life except animals. Thus, Hsp104 could represent an interesting drug target. Specific inhibition of Hsp104 activity might antagonize non-metazoan parasites that depend on a potent heat shock response, while producing little or no side effects to the host. However, no small molecule inhibitors of Hsp104 are known except guanidinium chloride. Here, we screen over 16,000 small molecules and identify 16 novel inhibitors of Hsp104 ATPase activity. Excluding compounds that inhibited Hsp104 activity by non-specific colloidal effects, we defined Suramin as an inhibitor of Hsp104 ATPase activity. Suramin is a polysulphonated naphthylurea and is used as an antiprotozoal drug for African Trypanosomiasis. Suramin also interfered with Hsp104 disaggregase, unfoldase, and translocase activities, and the inhibitory effect of Suramin was not rescued by Hsp70 and Hsp40. Suramin does not disrupt Hsp104 hexamers and does not effectively inhibit ClpB, the *E. coli* homolog of Hsp104, establishing yet another key difference between Hsp104 and ClpB behavior. Intriguingly, a potentiated Hsp104 variant, Hsp104^A503V^, is more sensitive to Suramin than wild-type Hsp104. By contrast, Hsp104 variants bearing inactivating sensor-1 mutations in nucleotide-binding domain (NBD) 1 or 2 are more resistant to Suramin. Thus, Suramin depends upon ATPase events at both NBDs to exert its maximal effect. Suramin could develop into an important mechanistic probe to study Hsp104 structure and function.

## Introduction

For proteins to perform their biological function, folding into the appropriate three-dimensional shape is of paramount importance [1]. Protein misfolding can result in cellular toxicity and lead to catastrophic diseases, such as Parkinson disease, Huntington disease and amyotrophic lateral sclerosis [1]–[3]. Thus, cells have evolved sophisticated chaperone systems to promote successful protein folding and preserve proteostasis [4], [5]. While most chaperones act by preventing protein misfolding [5], Hsp104 is capable of reversing protein aggregation [3], [6]–[8].

Hsp104 is a member of the AAA+ family of ATPases and utilizes energy from ATP hydrolysis to dissolve disordered protein aggregates as well as amyloid fibers [3], [6], [8], [9]. It assembles into a homohexameric ring structure with a central channel [7]. Hsp104, and its bacterial homolog ClpB, drive protein disaggregation by directly translocating substrates through this channel [10]–[15]. Each Hsp104 monomer contains an N-terminal domain, two AAA+ nucleotide-binding domains (NBD1 and NBD2), a coiled-coil middle domain, and a C-terminal region required for hexamerization [16]. Both NBDs contain Walker A and Walker B motifs that are critical for nucleotide binding and hydrolysis, respectively [17]. Most ATP hydrolysis happens at NBD1, whereas NBD2 has a primarily nucleotide-dependent oligomerization function [18], [19].

Hsp104 hexamers adapt different mechanisms of intersubunit collaboration to disaggregate amorphous aggregates versus amyloid [9]. Remarkably, this molecular motor can remodel amyloid substrates alone, without the aid of any other chaperones [6], [9]. However, to remodel amorphous protein aggregates, Hsp104 needs to collaborate with the Hsp110, Hsp70 and Hsp40 chaperone system, and the small heat shock proteins Hsp26 and Hsp42 can enhance disaggregase activity further [8], [20]–[24]. In vitro, mixtures of ATP and ATPγS (a slowly hydrolyzable ATP analog) enable Hsp104 to dissolve amorphous aggregates in the absence of other chaperones [25].

Hsp104 is highly conserved in eubacteria and eukaryotes [23], [24]. Indeed, Hsp104 is essential for cell viability in challenging conditions when proteins tend to aggregate more readily [26], [27]. Animal cells do not have an Hsp104 homolog [23], [24]. Thus, Hsp104 is a promising drug target against a myriad of microorganisms. For instance, Hsp101, the Hsp104 homolog in the malaria parasite *Plasmodium falciparum* is essential for parasite survival and has become a prime drug target [28], [29]. Indeed, a small molecule Hsp104 inhibitor could potentially treat a great variety of infections. Moreover, such a small molecule could greatly aid in the study of the structural and mechanistic basis of Hsp104 activity. Not only would a small-molecule inhibitor provide a way to rapidly silence Hsp104, but it might also hold the key to stabilizing Hsp104 hexamer structure to achieve a crystal structure that has remained so elusive. However, only one small-molecule inhibitor of Hsp104 activity is known to date: guanidinium hydrochloride (GdmCl), which is effective at millimolar concentrations [30], [31]. High-throughput screening has led to small molecule inhibitors for other molecular chaperones such as Hsp70 and Hsp90, as well as other AAA+ proteins, including p97 and even ClpB [32]–[36]. Here, we employ a high-throughput screen of over 16,000 compounds and identify 16 novel inhibitors of Hsp104 ATPase activity. We then excluded small molecules that inhibit Hsp104 by non-specific colloidal mechanisms. Thus, we isolated Suramin as a robust inhibitor of Hsp104 ATPase and disaggregase activities. Suramin also interfered with the unfolding and translocation activities of Hsp104. Hsp104 inhibition by Suramin was not rescued by Hsp70 and Hsp40. Interestingly, Suramin cannot inhibit ClpB to the same extent as Hsp104, thus highlighting the functional differences between these two related proteins [9], [16], [37]. Suramin does not act by disrupting Hsp104 hexamers, but depends upon ATPase activity at NBD1 and NBD2 to exert its maximal effect on Hsp104.

## Materials and Methods

### Materials

All chemicals were purchased from Sigma Aldrich (St. Louis, MO) unless otherwise specified. Creatine kinase was purchased from Roche. Firefly luciferase and FITC-casein were purchased from Sigma Aldrich. Quantilum recombinant luciferase was purchased from Promega (Madison, WI). Hsp70 and Hsp40 were purchased from Enzo Life Sciences (Farmingdale, NY).

### Protein Expression and Purification

Hsp104 variants (Hsp104^WT^, HAP, Hsp104^DWB^, Hsp104^T317A^, Hsp104^A503V^ and Hsp104^N728A^) were purified as reported previously [37]–[39]. Briefly, untagged Hsp104 was transformed into BL21-DE3 RIL cells (Agilent Technologies, Santa Clara, CA). Expression was induced at an OD_600_ of 0.4–0.6 with 1 mM IPTG for 15–18 h at 15°C. Cells were harvested via centrifugation (4,000 rpm, 4°C, 20 min), resuspended in lysis buffer (50 mM Tris-HCl, pH 8.0, 10 mM MgCl_2_, 2.5% glycerol (w/v), 2 mM β-mercaptoethanol, 5 µM pepstatin A, and 1 Mini-EDTA free protease tablets per 50 mL (Roche Applied Science, Indianapolis, IN), and lysed by sonication. Cell debris was removed via centrifugation at 16,000 rpm at 4°C for 20 min. The supernatant was applied to Affi-Gel Blue resin (Bio-Rad Laboratories, Hercules, CA). Supernatant and resin were rotated at 4°C for 4 h. Resin was then washed with wash buffer (50 mM Tris-HCl, pH 8.0, 10 mM MgCl_2_, 100 mM KCl, 2.5% glycerol (w/v), 2 mM β-mercaptoethanol). Hsp104 was eluted with high-salt buffer (wash buffer plus 1 M KCl). Hsp104 was then further purified by ResourceQ anion exchange chromatography using running buffer Q (20 mM TrisHCl pH 8.0, 0.5 mM EDTA, 5 mM MgCl_2_, 50 mM NaCl) and eluted with a linear gradient of buffer Q+ (20 mM Tris-HCl pH 8.0, 0.5 mM EDTA, 5 mM MgCl_2_, 1 M NaCl). Eluate was exchanged into storage buffer (40 mM HEPES-KOH pH 7.4, 150 mM KCl, 20 mM MgCl_2_, 10% glycerol, 1 mM DTT), snap frozen and stored at −80°C. High salt storage buffer (40 mM HEPES-KOH pH 7.4, 500 mM KCl, 20 mM MgCl_2_, 10% glycerol, 1 mM DTT), was used for storage of Hsp104^A503V^. RepA_1–70_-GFP was purified as described [40]. The eluted protein was then used with the tag. GroEL_trap_ was purified as previously published [25]. C-terminally His-tagged ClpP was overexpressed in BL21(DE3) cells and purified using Ni Sepharose 6 Fast Flow following standard procedures. The eluted protein was concentrated and exchanged into 20 mM Tris-HCl pH 7.5, 100 mM KCl, 0.1 mM EDTA, 10% glycerol, and 5 mM DTT. ClpB was purified as described [41].

### High-Throughput Screening Assay Reagents

ATPase activity was assessed by the release of inorganic phosphate determined using a malachite green phosphate detection kit (Innova Biosciences, Cambridge, United Kingdom). Microtiter plates (384-well) were purchased from PerkinElmer (Waltham, MA). Three libraries were screened: The NIH Clinical Collection (450 small molecules), the Spectrum Collection (2,000 compounds, MicroSource, Gaylordsville, CT) and the HitFinder Collection (14,400 compounds, Maybridge).

### High-Throughput Hsp104 Plate Assay

We adapted the inorganic phosphate colorimetric assay to fit a 384-well plate format. A Ź-factor between 0.8 and 1 was calculated for the assay [42]. A 2X Hsp104 stock solution was dispensed into each well using a Biotek microflo (Biotek Instruments, Winooski, VT). Compounds were then transferred using a Janus 96/384 Modular Dispensing Tool (PerkinElmer). Finally, a 2X ATP solution was dispensed using the Biotek microflo. Final concentrations of Hsp104 (monomer) and ATP were 0.25 µM and 1 mM respectively. Seeking to avoid the discovery of competitive inhibitors, we designed our high-throughput screen to use a 100-fold excess of ATP with respect to the test compounds (10 µM each). Final buffer conditions were 20 mM HEPES-KOH pH 7.4, 20 mM NaCl and 10 mM MgCl_2_. The ATPase reaction was allowed to continue for 60 min, at which time the reaction was terminated by addition of the colorimetric reagent. Absorbance was measured at 635 nm on an EnVision Xcite Multilabel plate reader (PerkinElmer). Hits were tested in duplicate. Sixteen compounds were found to lower ATPase activity of Hsp104. Ten of these were subjected to a 6-point titration (40, 13.3, 4.44, 1.45, 0.49 and 0 µM) in the absence or presence of Triton X-100, BSA, or both Triton X-100 and BSA at the specified concentrations. Heat maps were created in Excel for Mac 2011 (Microsoft Corporation) using the Conditional Formatting feature (Color Scales).

### Manual ATPase Assay

Wild-type or mutant Hsp104 (0.25 µM monomer) in ATPase buffer (20 mM HEPES-KOH pH 7.4, 20 mM NaCl and 10 mM MgCl_2_) was incubated for 60 or 10 min at 25°C in the presence of ATP (1 mM) as noted. ATPase activity was assessed by the release of inorganic phosphate determined by using a malachite green phosphate detection kit (Innova). To ensure Suramin did not interfere with the colorimetric detection of free phosphate, Hsp104^WT^ (0.25 µM monomer) was incubated with ATP (1 mM) for 60 min and then different concentrations of Suramin were added to the reaction followed by the addition of the colorimetric reagents.

### Luciferase Disaggregation Assays

Luciferase reactivation was performed as described [8], [37]. To assemble aggregates, firefly luciferase (50 µM) in luciferase refolding buffer (LRB, 25 mM HEPES-KOH, 7.4, 150 mM KAOc, 10 mM MgAOc, 10 mM DTT) with 6 M urea was incubated at 30°C for 20 min. Luciferase was then rapidly diluted 100-fold into LRB, divided into 100 µL aliquots, snap frozen in liquid N_2_, and stored at −80°C. For reactivation assays, aggregated luciferase (50 nM) was incubated with Hsp104 (1 µM hexamer), plus 2.6 mM ATPγS, 2.6 mM ATP and an ATP regeneration system (1 mM creatine phosphate, 0.25 µM creatine kinase (Roche Applied Science)) for 90 minutes at 25°C. Luciferase assay system was purchased from Promega. Luciferase activity was assessed by luminescence measured on a Safire^2^ microplate reader (Tecan, Männedorf, Switzerland). The half maximal inhibitory concentration (IC_50_) was calculating by fitting luciferase-refolding data at different Suramin concentrations with Prism (GraphPad Software, Inc., La Jolla, CA).

### RepA Unfolding Assays

RepA_1–70_-GFP unfolding was performed as described previously [25]. Varying concentrations of Suramin (0, 25, or 100 µM) were included as noted.

### FITC-Casein Degradation Assays

FITC-casein (0.1 µM) was incubated with HAP (1 µM hexamer) and ClpP (21 µM monomer) at 25°C. ATP (5 µM) and an ATP-regenerating system were included in all reactions. Varying concentrations of Suramin (0, 25, or 100 µM) were included as noted. We monitored degradation of FITC-casein by measuring fluorescence (excitation 490 nm, emission 520 nm) using a Tecan Safire^2^ microplate reader (Tecan, Männedorf, Switzerland).

### Gel Filtration

Hsp104 was diluted into ATPase buffer with or without Suramin (100 µM) in the presence or absence of ATP (1 mM). The Hsp104 concentration was adjusted to 120 µM monomer and 20 µL of sample were fractionated by a Superdex 200 10/300 column (GE Healthcare Life Sciences, Piscataway, NJ) at room temperature and at a flow rate of 0.5 mL per minute in line with an Optilab T-rEX Refractive Interferometer (Wyatt Technologies, Santa Barbara, CA). Molecular weight markers were purchased from Bio-Rad Laboratories (Hercules, CA).

### Luciferase Activity Determination

Quantilum recombinant luciferase (native) was diluted to 5000, 500, 50, 5 and 0.5 nM in the presence or absence of Suramin. Dilutions were mixed with luciferase assay system (Promega). Luciferase activity was assessed by luminescence measured on a Safire^2^ microplate reader.

## Results and Discussion

### High-Throughput Screening for Small-Molecule Inhibitors of Hsp104 ATPase Activity

We aimed to identify small molecules capable of inhibiting Hsp104. To do so, we employed high-throughput screening using Hsp104 ATPase activity as a proxy for function. To detect changes in Hsp104 ATPase activity, we used a colorimetric assay detecting free inorganic phosphate (P_i_) based on the formation of a colored complex between P_i_ and a dye molecule under acidic conditions [43], [44]. P_i_ is produced from the Hsp104-driven hydrolysis of ATP. The colored complex concentration, a surrogate for P_i_ concentration and ATPase rate, is determined by measuring the absorbance of a sample at 635 nm. We used recombinant Hsp104 purified from *E. coli.* An Hsp104 mutant with mutations in both NBD1 and NBD2 Walker B sequence motifs (Hsp104^DWB^, carrying ATPase ablating mutations E285Q: E687Q) [45] was used as a negative control for ATPase activity [9]. Hsp104^WT^ activity results in a high A_635_ (high ATPase), while Hsp104^DWB^ activity results in a low A_635_ (low ATPase). We optimized the P_i_ colorimetric assay to fit a 384-well plate format. We selected the Hsp104 concentration (0.25 µM) on the basis of previous experience in our laboratory [9]. The ATP concentration was selected to be in a 100-fold excess of ATP with respect to the test compounds. Furthermore, we found that starting the reaction by the addition of ATP (as opposed to Hsp104) yielded the most reliable results; similarly, a reaction time of 60 minutes (compared to 10 minutes, 30 minutes and overnight) maximized the response for the assay (data not shown). We screened 16,850 compounds from the NIH Clinical Collection as well as the Spectrum and Maybridge compound libraries. Sixteen compounds were found to inhibit the ATPase activity of Hsp104 (**Table 1**). For instance, hexachlorophene and tannic acid inhibit Hsp104 ATPase activity by ∼80%, while Merbromin and Sennoside a inhibit by ∼20% (**Table 1**).

**Table 1:** Sixteen molecules inhibit hydrolysis of ATP by Hsp104.

Molecule name	% Inhibition
Hexachlorophene	80.21±1.00
Tannic acid	79.79±0.29
Cisplatin	76.58±1.06
Carboplatin	73.75±1.41
Theaflavin monogallates	57.25±7.19
Suramin	50.83±2.83
Gossypol-acetic acid complex	47.92±1.30
Hematein	45.71±5.95
Gossypol	42.33±4.83
Chlorophyllide Cu Complex Na Salt	33.79±2.18
Methacycline hydrochloride	33.33±2.12
Sucralfate Sodium (10% w/v in DMSO)	25.67±2.59
Aurin tricarboxylic acid	24.25±1.41
Epigallocatechin 3,5-digallate	21.88±2.53
Merbromin	21.17±3.54
Sennoside a	19.92±7.07

Hsp104 (0.25 µM monomer) was incubated for 60 min with ATP (1 mM) plus 16,850 small-molecule drugs (10 µM). The amount of P_i_ produced was measured by absorbance at 635 nm. Absorbance values were normalized to the absorbance produced by Hsp104 in the absence of the small molecules. Sixteen molecules were found to reproducibly interfere with Hsp104 ATPase activity. Inhibition values represent mean ±S.D. (*n* = 2).

**Figure 1:**
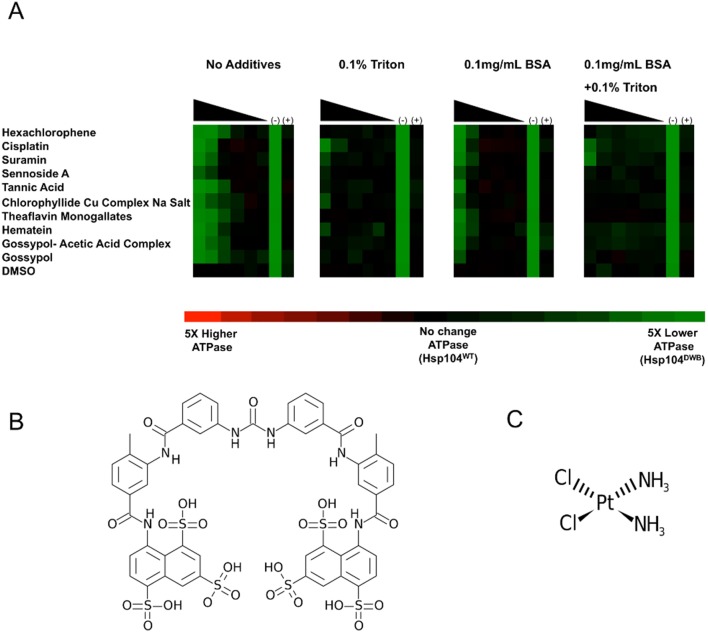
Suramin and Cisplatin inhibit Hsp104 ATPase activity even in the presence of detergent and BSA. (**A**) Hsp104 (0.25 µM monomer) was incubated for 60 min with ATP (1 mM) and varying concentrations (40, 13.3, 4.44, 1.45, 0.49 and 0 µM) of the noted molecules in the presence of Triton X-100 and BSA in the specified concentrations and combinations. The amount of P_i_ produced was measured by absorbance at 635 nm. Raw (non-normalized) absorbance values were transformed to a color scale, where the average absorbance produced by Hsp104^WT^ is represented as black. The color red represents absorbance values five-fold higher than average absorbance for Hsp104^WT^. Green represents absorbance values five-fold lower than the average absorbance obtained for Hsp104^WT^. Hsp104^DWB^ was used as a negative control (−). Hsp104^WT^ in the absence of inhibitors was used as a positive control (+). (**B, C**) Chemical structures of Suramin (**B**) and Cisplatin (**C**).

Several of the small molecules uncovered by our screen contained catechol groups, which are frequently a feature of promiscuous compounds in biochemical high throughput screens [46]. To exclude inhibition by non-specific colloidal effects [47], [48], we performed a dilution series for 10 out of the 16 inhibitors in the presence of: (1) Triton X-100, (2) BSA, and (3) both BSA and Triton X-100 (**Figure S1**). A common mechanism behind false-positive inhibition is the formation of promiscuous aggregates by self-assembly of small organic molecules in aqueous solution [47], [48]. These aggregates bind proteins and non-specifically inhibit their activity [47], [48]. By counter-screening in the presence of detergent, we can exclude small molecules that are likely to be inhibiting via the formation of aggregates as these are sensitive to detergent [49]. To further exclude non-specific interactions, we chose to use BSA, which has been proposed as an alternative to ionic detergents in systems where detergents are not well tolerated [49]. The presence of 0.1% Triton X-100 relieved inhibition of ATP hydrolysis by many of the small molecules (**Figure 1A**, second column from the left). BSA, at a concentration of 0.1 mg/mL, also appeared to lessen the effect of the inhibitors, though to a smaller extent than Triton X-100 (**Figure 1A**, third column from the left). Out of our initial ten inhibitors, we found Suramin and Cisplatin to most significantly inhibit Hsp104 ATPase activity in the presence of both Triton X-100 and BSA (**Figure 1A**, right column).

Suramin, an FDA-approved drug, is a symmetrical polysulfonated naphthylamine urea derivative (**Figure 1B**) [50]. This drug was developed more than one hundred years ago as a treatment for human African sleeping sickness [51]. More recently, Suramin has been shown to be an effective anticancer agent [52] and to reverse several autism-related features in a mouse model of the disease [53], [54]. Interestingly, Suramin and its derivatives have been shown to reduce the levels of prion protein in infected cells [55] by inducing its aggregation at the cell surface and thus inhibiting prion replication [56]. Suramin has a strong affinity for a variety of proteins and enzymes [57]; however, despite being a promiscuous inhibitor, it does not inhibit its targets by colloidal effects [58]. Suramin is also known to inhibit pyruvate kinases; its phenyl sulfonate groups bind their active sites in place of ATP [59]. Lastly, Suramin is known to inhibit the ATPase activity of RecA, a DNA-dependent ATPase involved in DNA repair [60].

**Figure 2:**
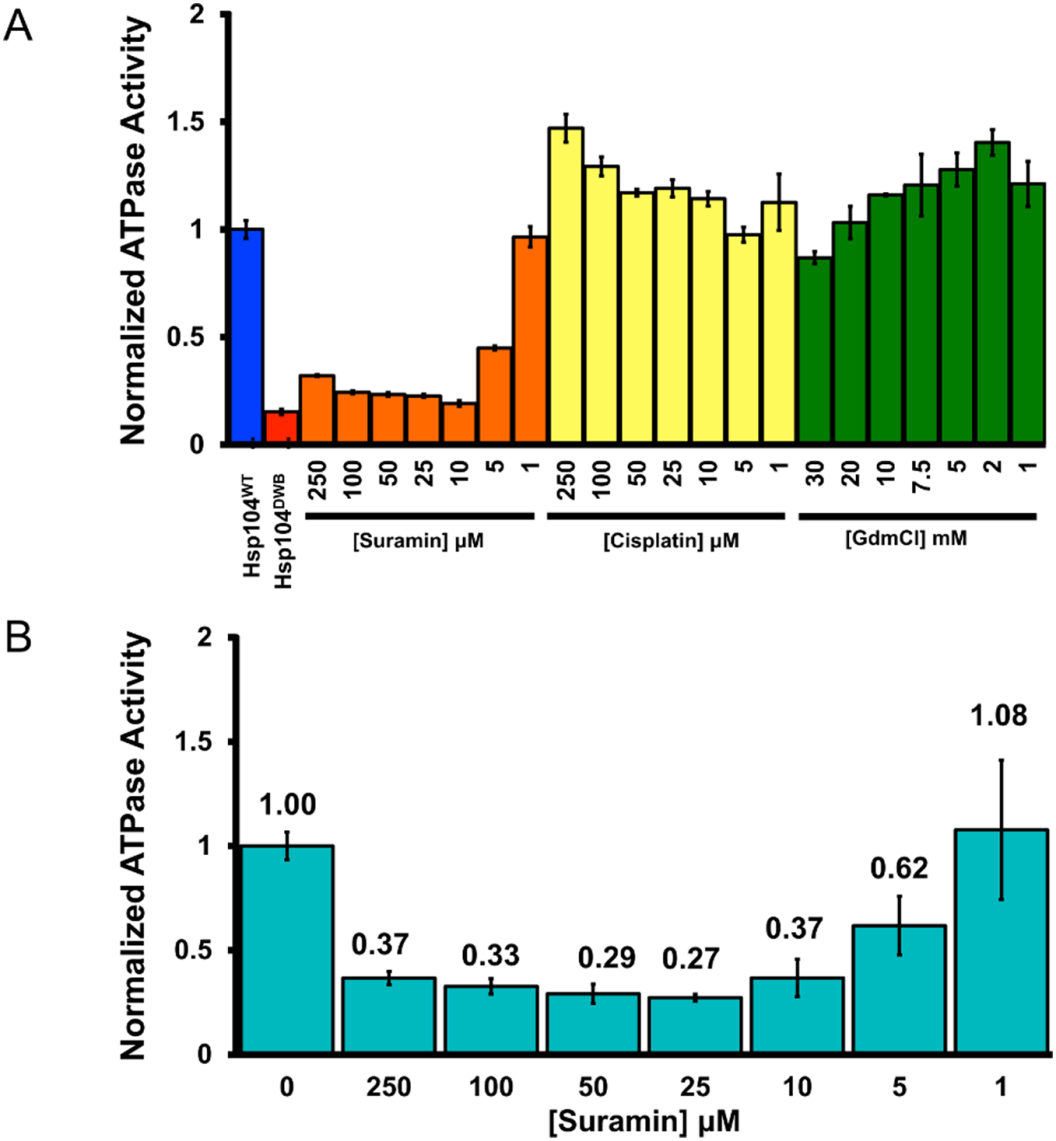
Suramin, but not Cisplatin, greatly inhibits Hsp104 ATPase activity. (**A**) Hsp104 (0.25 µM monomer) was incubated for 60 min with ATP (1 mM) and varying concentrations of Suramin, Cisplatin and Guanidinium Chloride (GdmCl). The amount of P_i_ produced was measured by absorbance at 635 nm. Raw absorbance values were normalized to the average absorbance yielded by Hsp104^WT^ in the absence of inhibitor. Values represent mean ±S.D. (*n* = 6). (**B**) Hsp104 (0.25 µM monomer) was incubated for 10 min with ATP (1 mM) and varying concentrations of Suramin. Raw absorbance values were normalized to the average absorbance yielded by Hsp104^WT^ in the absence of inhibitor. Values represent mean ±S.D. (*n* = 6).

Cisplatin (**Figure 1C**, cisplatinum or *cis*-diamminedichloroplatinum (II)), also an FDA-approved drug, is a highly effective chemotherapeutic agent used to treat several types of cancer including ovarian, testicular, penile, cervical, lung and bladder cancers [61]. It interacts with DNA to form DNA adducts which activate several signal transduction pathways and culminate in the activation of apoptosis [62]. Cisplatin can also form protein adducts by covalent modification of cysteine and methionine residues, which can even induce protein crosslinking and aggregation [63]. Curiously, we were unable to replicate the inhibitory effect of Cisplatin outside of the 384-well format (**Figure 2A**, yellow bars). Hence, we did not pursue Cisplatin any further.

### Suramin Inhibits Hsp104 ATPase Activity More Effectively Than GdmCl

We first set out to validate the effect of different concentrations of Suramin on the amount of ATP hydrolysis by Hsp104. In the high-throughput assay, we measured the amount of P_i_ produced by Hsp104-mediated ATP hydrolysis after 60 minutes. We scaled up the volume of our reactions for manual validation, but kept all other conditions the same. Under manual assay conditions, GdmCl a well documented inhibitor of Hsp104 ATPase activity [30], [31], [64], [65], only very mildly inhibited Hsp104 ATPase activity at the highest concentration tested (30 mM; **Figure 2A**, green bars). This finding is consistent with previous studies that found a large fraction of Hsp104 ATPase activity remained even at GdmCl concentrations of 100 mM [30]. By contrast, Suramin greatly diminished the amount of P_i_ produced in a concentration-dependent manner and was effective at micromolar concentrations (**Figure 2A**, orange bars). We confirmed Suramin did not interfere with the colorimetric detection of free phosphate (data not shown). To assess the effect of Suramin on the initial rate of ATP hydrolysis by Hsp104, we allowed the ATPase reaction to proceed for only 10 minutes in the presence of varying concentrations of Suramin (**Figure 2B**). As before, Suramin greatly diminished the amount of P_i_ produced in a concentration-dependent manner and was effective at micromolar concentrations (**Figure 2B**). It is important to note, however, that the ATPase activity of Hsp104 was not completely ablated for any of the Suramin concentrations tested (**Figure 2B**). Based on these data, we calculated the half maximum inhibitory concentration (IC_50_) of Suramin to be ∼3.39 µM.

### Suramin Inhibits Hsp104 Refolding, Unfolding, and Translocation Activities

**Figure 3:**
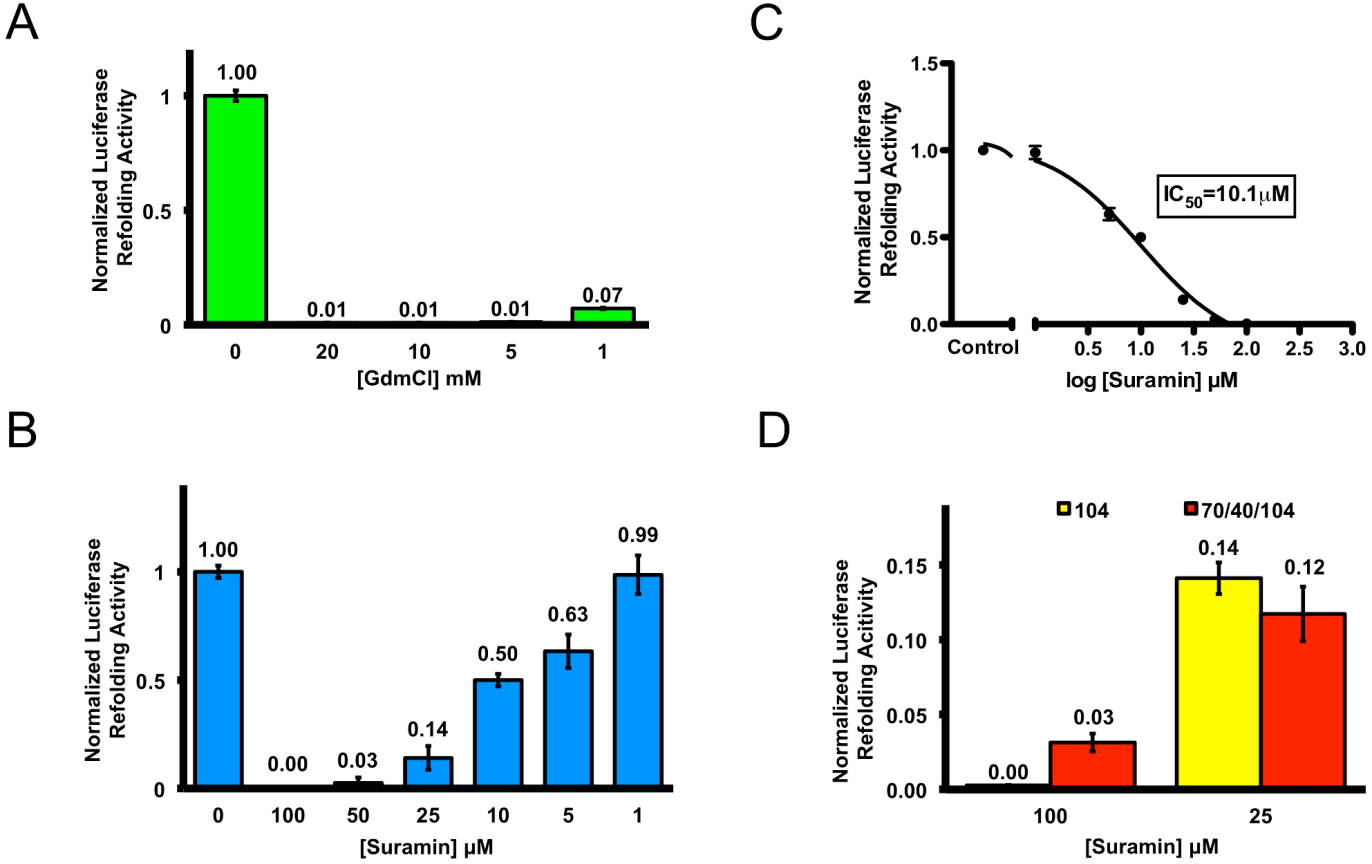
Suramin greatly inhibits Hsp104 refolding activity. (**A**) Urea-denatured firefly luciferase aggregates were incubated for 90 min at 25°C with Hsp104 (1 µM hexamer) plus 1∶1 mixtures of ATP and ATPγS and varying concentrations of GdmCl. Luciferase reactivation was then determined and converted to % WT disaggregase activity in the presence of ATPγS: ATP. Values represent mean ±S.D. (*n* = 3–6). (**B**) Reactions were performed as in (**A**) except varying concentrations of Suramin were used. Values represent mean ±S.D. (*n* = 6–10). (**C**) Half maximal inhibitory concentration (IC_50_) for Suramin-mediated inhibition of Hsp104. IC_50_ was calculating by fitting luciferase-refolding data at different Suramin concentrations. (**D**) Reactions were performed as in (**A**) except that 1∶1 mixtures of ATP and ATPγS were replaced with Hsp70 (1 µM) and Hsp40 (1 µM) plus ATP. The Hsp104 alone condition (yellow bars) was carried out with 1∶1 mixtures of ATP and ATPγS as in (**A**). Each condition is normalized to the refolding activity in each corresponding condition in the absence of inhibitor.

To assess Hsp104 function, the capacity of Hsp104 to refold amorphous protein aggregates was determined by measuring the amount of urea-denatured luciferase that could be reactivated by Hsp104 alone or in the presence of inhibitors. The activity of refolded luciferase is quantified by luminescence and then used as a proxy for Hsp104 refolding activity. To bypass the requirement for other chaperones, we used a 1∶1 ratio of ATP to ATPγS to activate Hsp104 [25]. Despite only having a modest effect on Hsp104 ATPase activity (**Figure 2A**), all concentrations of GdmCl tested very strongly inhibited the luciferase reactivation activity of Hsp104 (**Figure 3A**). Importantly, these GdmCl concentrations do not inhibit firefly luciferase refolding by other molecular chaperones [66], indicating a very strong inhibition of Hsp104 disaggregase activity as noted previously [8], [67]. Thus, GdmCl grossly perturbs Hsp104 disaggregase activity without having an equivalent effect on Hsp104 ATPase activity (e.g. at 1 mM GdmCl). This disproportionate inhibitory effect of GdmCl on Hsp104 disaggregase activity compared to ATPase activity suggests that the major effect of GdmCl is to uncouple Hsp104-catalyzed ATP hydrolysis from protein disaggregation. Suramin, at a concentration of 100 µM, completely ablated the ability of Hsp104 to refold denatured luciferase, whereas 5 mM GdmCl was needed to elicit the same level of inhibition (**Figure 3A, B**). This effect was gradually reduced with decreasing concentrations of Suramin (**Figure 3B**). We calculated Suramin to have an IC_50_ of ∼10.1 µM (**Figure 3C**). Unlike GdmCl (3 mM), Suramin (100 µM) impaired neither thermotolerance nor [*PSI^+^*] propagation in Δ*pdr5* yeast (which lack Pdr5, an ABC transporter that expels small molecules from the cell [68]), two activities that absolutely require Hsp104 (data not shown) [26], [27], [65], [69]. This lack of activity in vivo is likely due to poor uptake by yeast cells or titration by other Suramin-binding proteins. Indeed, Suramin is known to interact strongly with many proteins [57], [70].

Despite the lack of in vivo activity, Suramin could still serve as a useful mechanistic probe to study Hsp104 function in vitro. Intriguingly, inhibition by Suramin was not rescued by inclusion of Hsp70 and Hsp40 in the disaggregation reaction (**Figure 3D**, red bars). As a control, we verified that Suramin did not inhibit luciferase activity itself. At the luciferase concentration used in our refolding assay (50 nM), Suramin (100 µM) did not inhibit luciferase activity (data not shown). These data suggest that the observed reduction in luciferase refolding is most likely due to a reduction in Hsp104 activity.

**Figure 4:**
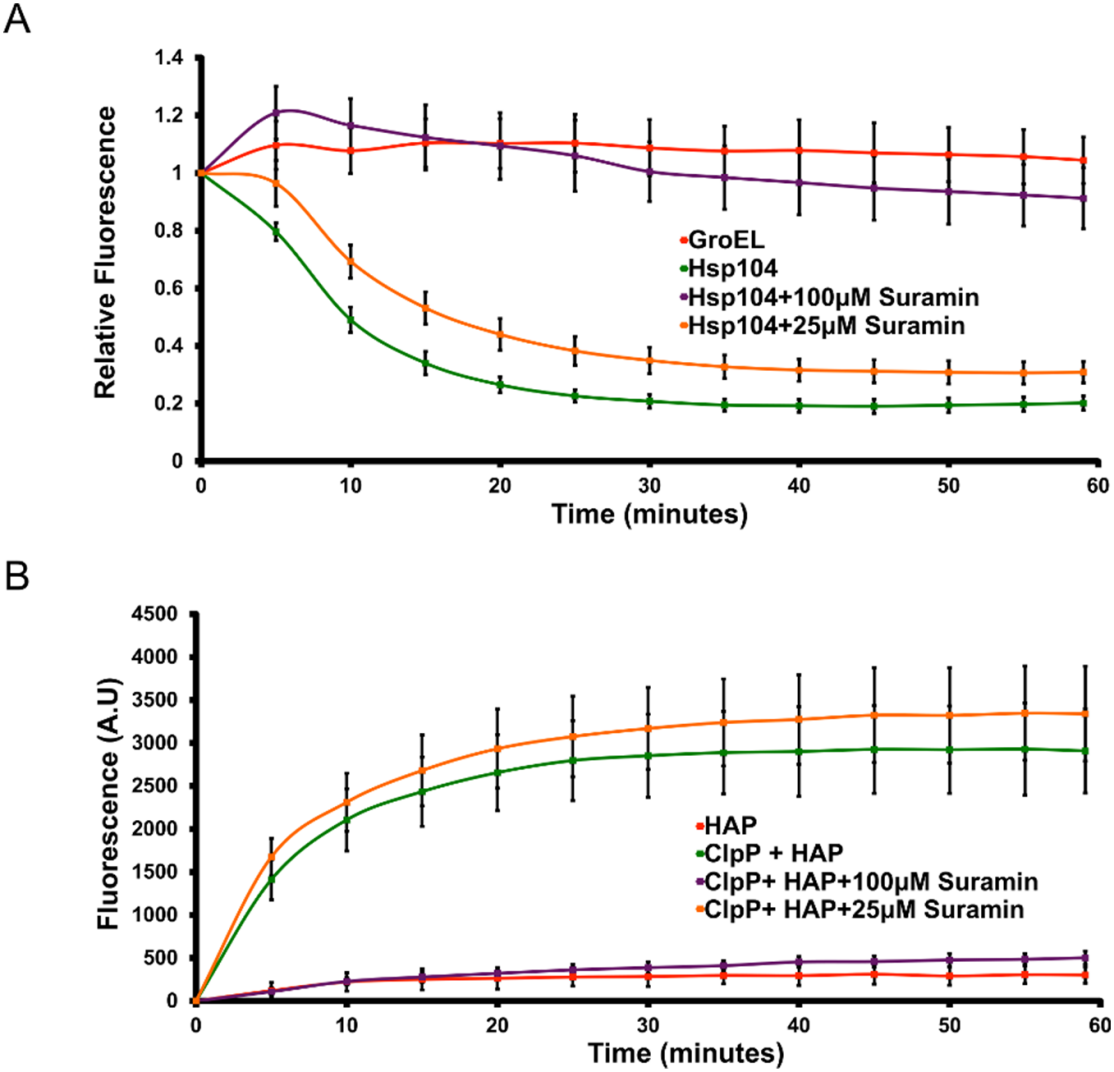
Suramin inhibits Hsp104-mediated substrate unfolding and translocation. (**A**) RepA_1–70_-GFP was incubated with Hsp104^WT^ and GroEL_trap_ plus a 1∶1 mixture of ATP and ATPγS. GFP unfolding was measured by fluorescence. GroEL_trap_ alone is shown as a negative control (red line). Fluorescence values were normalized to initial raw fluorescence for each sample. Values represent mean ±S.D. (*n* = 3). (**B**) FITC-casein (0.1 µM) was incubated at 25°C with HAP (1 µM hexamer) and ClpP (21 µM monomer) plus ATP (5 mM) and an ATP regeneration system. Degradation of FITC-casein was monitored by fluorescence. Initial fluorescence was subtracted from raw fluorescence values for each sample. HAP alone is shown as a negative control (red line). Values represent mean ±S.D. (*n* = 5).

Next, we determined that Suramin hinders the unfoldase activity of Hsp104 using a RepA_1–70_-GFP substrate (**Figure 4A**). To assess RepA_1–70_-GFP unfolding without interference from spontaneous refolding, we added GroEL_trap_, which captures unfolded proteins to prevent refolding [25], [71]. Suramin, at a concentration of 100 µM, prevented substrate unfolding (**Figure 4A**, purple line). By contrast, unfolding was only mildly inhibited by 25 µM Suramin (**Figure 4A**, orange line).

Next, we established that Suramin hampers substrate translocation by Hsp104 using an engineered Hsp104 variant, HAP, which anchors to the bacterial peptidase ClpP to form a novel proteolytic system [13]. Thus, HAP translocates fluorescein isothiocyanate (FITC)-casein for degradation by ClpP. Fluorescence increases as FITC is released from degraded casein and can be used as a surrogate for substrate translocation [40]. Suramin, at a concentration of 100 µM, completely abolished substrate translocation (**Figure 4B**, purple line). In contrast, treatment with 25 µM inhibitor permits translocation activity equivalent to that by untreated HAP in conjunction with ClpP (**Figure 4B**, orange and green lines). Note that both the unfolding and translocation activities appear less sensitive to Suramin than disaggregase activity (**Figures 3B** **,** **4A and 4B**). Thus, disaggregation of protein aggregates makes more stringent demands on the Hsp104 hexamer than the unfolding or translocation of soluble substrates. Altogether, we have established that Suramin greatly decreases Hsp104 ATPase activity, drastically impairs its capacity to refold luciferase and hinders its substrate unfolding and translocation activities.

### ClpB Displays Resistance to Suramin

**Figure 5:**
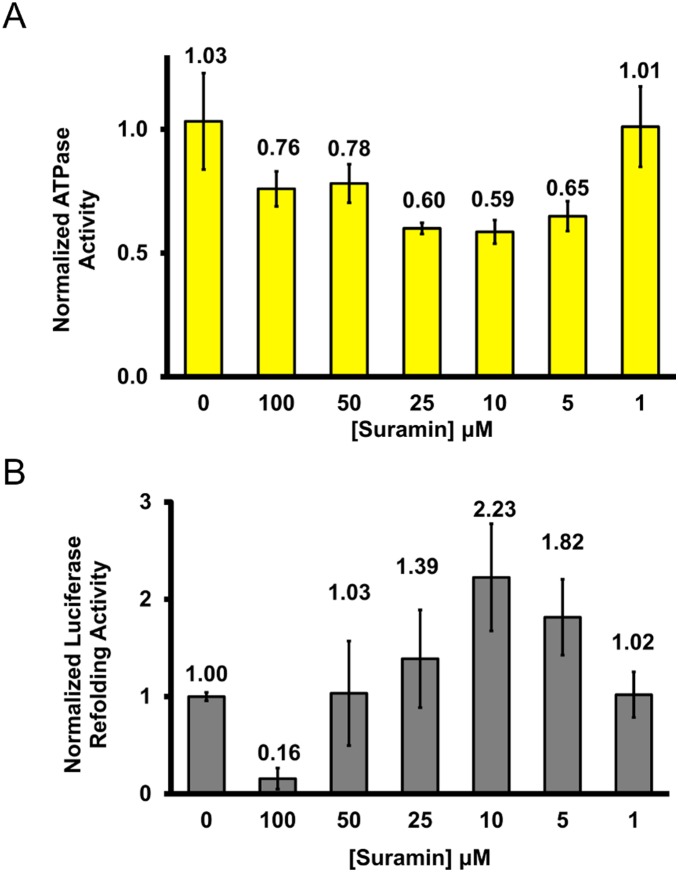
ClpB displays resistance to Suramin. (**A**) ClpB (0.25 µM monomer) was incubated for 10 min with ATP (1 mM) and varying concentrations of Suramin. The amount of P_i_ produced was measured by absorbance at 635 nm. Absorbance values were normalized to the absorbance produced by ClpB in the absence of inhibitor. Values represent mean ±S.D. (*n* = 6). (**B**) Urea-denatured firefly luciferase aggregates were incubated for 90 min at 25°C with ClpB (1 µM hexamer) in the presence of 1∶1 mixtures of ATP and ATPγS and varying concentrations of Suramin. Reactivation of luciferase was then determined by luminescence and converted to % WT ClpB activity (activity of 1 µM WT ClpB in the presence of ATP and ATPγS). Values represent mean ±S.D. (*n = *6–12).

We also assessed the effect of Suramin on ClpB, the *E. coli* homolog of Hsp104. Curiously, ClpB ATPase activity is not greatly hindered by Suramin even at high concentrations (**Figure 5A**). For instance, 100 µM Suramin inhibits over 60% of Hsp104 ATPase activity, while the same concentration only inhibits 24% of ClpB ATPase activity (**Figure 2B** **and** **5A**). Next, we assessed ClpB-mediated refolding of chemically-denatured luciferase aggregates in the presence of Suramin. As before, we bypassed the use of co-chaperones by using a 1∶1 ratio of ATP to ATPγS to stimulate ClpB, in the absence of DnaK, DnaJ and GrpE (Hsp70, Hsp40 bacterial homologs and a nucleotide exchange factor, respectively) [25]. Only high concentrations of Suramin (100 µM) drastically inhibited the ClpB-mediated refolding of luciferase (**Figure 5B**). Nevertheless, 16% of ClpB disaggregase activity remains even at this concentration. Indeed, ClpB is much less sensitive to Suramin than Hsp104; for instance, at a Suramin concentration of 50 µM, ClpB retains 100% of its refolding power (**Figure 5B**), while Hsp104 is practically inactive (**Figure 3B**). Importantly, this result confirms that Suramin is not inhibiting the refolding of luciferase itself. Strikingly, low concentrations of Suramin increased the amount of luciferase ClpB was able to reactivate (**Figure 5B**). The divergent behavior of Hsp104 and ClpB in response to Suramin further supports the notion that these two machines function by different mechanisms despite the similarities in their sequence and architecture [9]. Indeed, functional differences between ClpB and Hsp104 abound. For instance, NBD1 is primarily responsible for ATPase activity in Hsp104 [18], [39], [72], [73], whereas both NBDs contribute in ClpB [74]. Furthermore, nucleotide binding to NBD1 is essential for ClpB hexamerization [74]; in contrast, nucleotide binding to NBD2 is needed for Hsp104 to hexamerize [18], [72], [75]. Hsp104 and ClpB even diverge in their mechanisms of collaboration with Hsp70 [37]. Most interestingly, Hsp104 is able to rapidly process amyloid substrates while ClpB cannot [6], [9], [76]. In addition to these functional disparities, recent data draws attention to potential structural differences between these proteins. Cryo-electron microscopy structures of the BAP variant of ClpB (which binds ClpP analogously to HAP) are not compatible with some of the disulfide bonds engineered in yeast Hsp104 [37], [77]. Our results with Suramin further support the existence of operational differences between ClpB and Hsp104.

### Suramin Does Not Disrupt Hsp104 Hexamers

**Figure 6:**
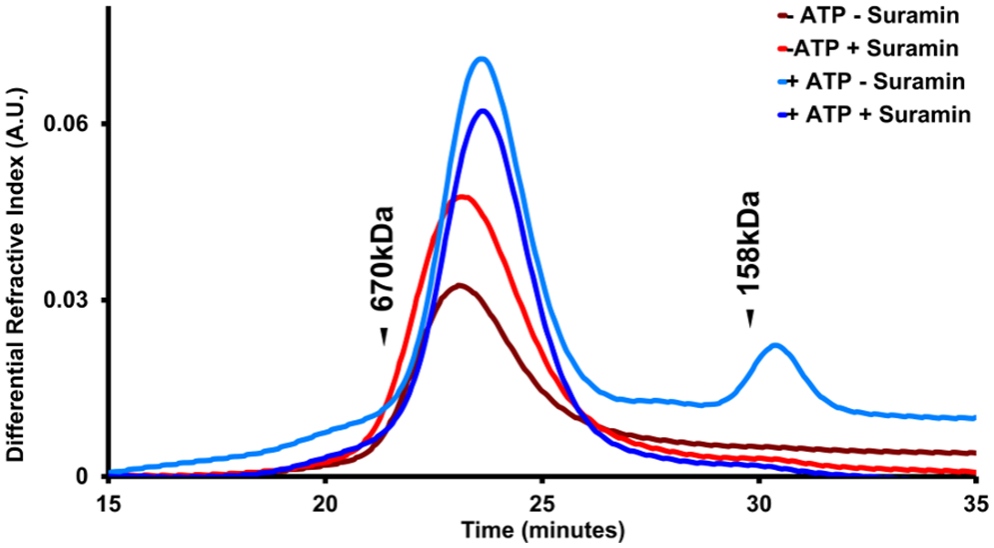
Suramin does not disrupt Hsp104 hexamers. Gel filtration analysis of Hsp104 in the absence and presence of Suramin (100 µM) both in the presence and absence of ATP (1 mM). Protein elution profiles monitored by refractive index are shown for Hsp104 with and without nucleotides and in the absence or presence of Suramin in the sample and running buffer. Elution positions of relevant molecular weight standards are shown.

Since Suramin is known to interfere with the oligomerization of proteins [78], we asked whether it might disrupt Hsp104 hexamers. Hsp104 must exist as a hexamer to hydrolyze ATP and perform its functions [72], [79], and thus a small molecule hindering its oligomerization would inhibit activity. **Figure 6** shows gel filtration elution profiles of Hsp104 with or without ATP in the absence or presence of Suramin (100 µM). Without nucleotide or inhibitor, Hsp104 eluted as a broad peak with an apparent size of ∼600 kDa (**Figure 6**, dark red line). Interestingly, the presence of Suramin did not significantly change the elution profile, demonstrating that the inhibitor does not interfere with hexamerization (**Figure 6**, red line). When 1 mM ATP is present in the sample and running buffer, the majority of Hsp104 still eluted as a hexamer; however, a smaller peak corresponding to monomeric Hsp104 appears (**Figure 6**, light blue line). The fraction of Hsp104 eluting as monomers in the presence of ATP could be due to ATP hydrolysis by Hsp104 on the column. Consistent with this idea, when Suramin is added and ATP hydrolysis is inhibited, only the ∼600 kDa peak is observed (**Figure 6**, dark blue line). From these data, we conclude that Suramin does not disrupt Hsp104 hexamers, and thus must inhibit the activity of hexameric Hsp104.

### A Potentiated Hsp104 Variant Is Particularly Sensitive to Suramin

**Figure 7:**
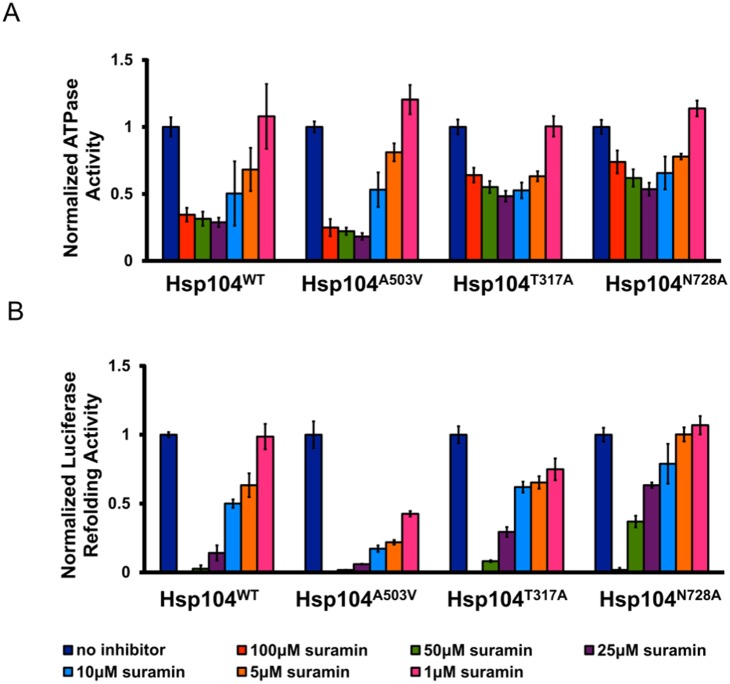
Hsp104 variants respond differently to Suramin than Hsp104^WT^. (**A**) Hsp104 variants (0.25 µM monomer) were incubated for 10 min with ATP (1 mM) and varying concentrations of Suramin. The amount of Pi produced was measured by absorbance at 635 nm. Absorbance values were normalized to the absorbance produced by each Hsp104 variant in the absence of inhibitor. Values represent mean ±S.D. (*n* = 6). (**B**) Urea-denatured firefly luciferase aggregates were incubated for 90 min at 25°C with Hsp104 variants (1 µM hexamer) plus 1∶1 mixtures of ATP and ATPγS (or ATP alone) and varying concentrations of Suramin. Luciferase reactivation was then determined and normalized to untreated disaggregase activity for each variant in the presence of ATPγS: ATP (or ATP alone). 1∶1 mixtures of ATP and ATPγS were used for Hsp104^WT^ and Hsp104^T317A^, while ATP alone was used for Hsp104^A503V^ and Hsp104^N728A^. Values represent mean ±S.D. (*n* = 6–12).

To learn more about how Suramin interacts with Hsp104, we examined its effect on a hyperactive variant of Hsp104, Hsp104^A503V^ [40], [80], [81]. This mutation is located in the middle domain and is thought to weaken autoinhibitory interactions that diminish Hsp104 activity or to induce conformational changes that mimic an allosteric activation of the protein [40]. The A503V mutation circumvents the need for Hsp70 and Hsp40 in remodeling amorphous aggregates (even in the absence of ATPγS) and displays increased ATPase activity, substrate translocation speed, unfoldase activity, and amyloid disaggregase activity [40]. Resembling our results for Hsp104^WT^, Suramin reduced Hsp104^A503V^ ATPase activity in a concentration-dependent manner (**Figure 7A**). Both Hsp104 and Hsp104^A503V^ ATPase are maximally inhibited at an inhibitor concentration of 25 µM. However, Hsp104^A503V^ is slightly more sensitive to inhibition. For example, at 25 µM Suramin, Hsp104^WT^ has an ATPase activity of 30% (relative to uninhibited Hsp104) while Hsp104^A503V^ has an activity of 18% (**Figure 7A**).

Intriguingly, we found Hsp104^A503V^ luciferase reactivation activity to be considerably more sensitive to Suramin than that of Hsp104^WT^ (**Figure 7B**). While Hsp104^WT^ has a 100% refolding activity in the presence of 1 µM Suramin, Hsp104^A503V^ has less than 50% activity at the same inhibitor concentration (**Figure 7B**). Based on refolding data, the IC_50_ for Hsp104^A503V^ is ∼0.61 µM, which is much lower than that for Hsp104^WT^ (IC_50_∼10.1 µM). These observations further suggest that Hsp104^A503V^ hexamers are regulated differently than Hsp104^WT^ [40]. Suramin appears to be exploiting this difference to preferentially inhibit the potentiated variant.

### Suramin Preferentially Inhibits Disaggregase Activity Catalyzed by ATP Hydrolysis at NBD2

To evaluate the effect of Suramin on individual NBDs, we took advantage of the AAA+ sensor-1 mutants T317A and N728A [39]. Hsp104^T317A^ and Hsp104^N728A^ can bind ATP but are unable to hydrolyze it at NBD1 and NBD2 respectively [39]. If, for instance, Suramin acts preferentially on NBD1, we would expect Hsp104^T317A^ to be resistant to Suramin. On the other hand, if Suramin acts on NBD2 then we would expect Hsp104^N728A^ to be unaffected.

Suramin inhibited Hsp104^T317A^ and Hsp104^N728A^ ATPase activity in a dose-dependent manner (**Figure 7A**). However, the extent of inhibition was much lower for both Hsp104^T317A^ and Hsp104^N728A^ compared to Hsp104^WT^ and Hsp104^A503V^. For instance, at 100 µM Suramin, Hsp104^WT^ ATPase activity is ∼35% of that of the uninhibited protein, whereas Hsp104^T317A^ and Hsp104^N728A^ exhibit rates of ∼65% and ∼75% respectively (**Figure 7A**). As these two mutants are only able to hydrolyze ATP at one NBD, they do not “cycle” through hydrolysis events at both NBDs [39], unlike Hsp104^WT^ and Hsp104^A503V^. These findings suggest that maximal inhibition by Suramin depends on both NBD1 and NBD2 being able to hydrolyze ATP. Indeed, Hsp104^A503V^ cycles through more hydrolysis events at both NBDs than Hsp104^WT^ [40], [82], [83] and is even more sensitive to Suramin (**Figure 7A**). Nonetheless, Suramin inhibits the ATPase activities of both Hsp104^T317A^ and Hsp104^N728A^ to a similar extent, indicating that it can inhibit the global ATPase activity of Hsp104, including ATPase reactions occurring at both NBDs. It is important to note that Suramin does not preferentially inhibit NBD1 or NBD2 ATPase activity. This finding differentiates Suramin from GdmCl, which inhibits the ATPase activity of NBD1 but not NBD2 of Hsp104 [84].

We found both Hsp104^T317A^ and Hsp104^N728A^ luciferase refolding activities to be inhibited by Suramin in a concentration-dependent manner (**Figure 7B**). Akin to our results for ATPase activity, the extent of the inhibition was much lower for both Hsp104^T317A^ and Hsp104^N728A^ compared to Hsp104^WT^. Thus, we find both sensor-1 mutants are more refractory to Suramin than Hsp104^WT^ (**Figure 7B**). This result further reinforces that Suramin is not inhibiting the refolding of luciferase after it is released from Hsp104. Luciferase reactivation by Hsp104^N728A^ is more resistant to Suramin, revealing an inhibitor preference for protein disaggregation catalyzed by ATP hydrolysis at NBD2. For instance, at 50 µM Suramin, Hsp104^N728A^ retains ∼36% refolding activity, while Hsp104^T317A^ is approximately 8% active (**Figure 7B**). Likewise, Hsp104^N728A^ retains ∼100% refolding activity in the presence of 1 µM Suramin, while Hsp104^T317A^ has approximately 75% activity at the same inhibitor concentration. These findings suggest that Suramin uncouples ATP hydrolysis from protein disaggregation more effectively at NBD2 than NBD1. Hence, we conclude that Suramin preferentially inhibits disaggregase activity catalyzed by ATP hydrolysis at NBD2. Based on refolding data, the IC_50_ values for Hsp104^T317A^ and Hsp104^N728A^ are ∼20.5 µM and ∼92.4 µM respectively, which are much higher than that for Hsp104^WT^ (∼10.1 µM) and Hsp104^A503V^ (∼0.61 µM). These results suggest that Suramin more effectively inhibits Hsp104 when it cycles between ATP hydrolysis events at NBD1 and NBD2, as the sensor-1 mutants primarily hydrolyze ATP at one NBD and are less susceptible to the molecule than Hsp104^WT^.

Hsp104 NBD1 and NBD2 have been proposed to adopt two distinct conformations (relaxed, R, and tense, T) that are reciprocally regulated by multiple allosteric pathways [17]. The tense conformation has low activity, while the relaxed conformation is highly active. It is proposed that upon ATP binding to NBD1, Hsp104 switches from a less active NBD1 [R] NBD2 [T] state to a highly active NBD1 [T] NBD2 [R] state [17]. It is possible that Suramin retains NBD2 in a [T] conformation and hinders its transition to an active conformation. Thus, Hsp104 would be stuck in an NBD1 [T] NBD2 [T] state or returned to NBD1 [R] NBD2 [T]. This might help explain why Hsp104^N728A^ is much less sensitive to Suramin, as this NBD2 transition is already disrupted by mutation. Altogether, our findings suggest that Suramin preferentially disrupts disaggregation driven by ATP hydrolysis at NBD2.

## Conclusions

We performed the first high throughput screen for Hsp104 ATPase inhibitors, encompassing over 16,000 small-molecule compounds. We found 16 molecules that inhibit Hsp104 ATPase activity in vitro. Of these, Suramin hinders the rate of ATPase activity in a specific, non-colloidal manner. Suramin also inhibits Hsp104 disaggregase, unfoldase, and translocase activities. Suramin inhibits Hsp104 without disrupting the oligomerization state of the disaggregase. Suramin-mediated inhibition of Hsp104 is not rescued by Hsp70 and Hsp40. Intriguingly, ClpB is much less sensitive to Suramin than Hsp104, which supports prior observations that these homologs function by different mechanisms. A potentiated variant of Hsp104 proved to be more sensitive to Suramin than the wild-type protein. Variants defective in ATP hydrolysis revealed a preference for Suramin to inhibit disaggregase activity catalyzed by NBD2 over NDB1. Overall, our data suggests Suramin takes advantage of Hsp104 “cycling” between ATP hydrolysis events at NBD1 and NBD2 to exert its maximal inhibitory effects. Future experiments will delineate the precise mechanism by which Suramin engages Hsp104 and exerts its inhibitory effects. We hope that Suramin will greatly aid in the study of the molecular mechanisms underlying Hsp104 function.

## Supporting Information

Figure S1 Small Molecules that Inhibit Hsp104 ATPase Activity. Chemical structures and common names are shown for nine molecules found to inhibit Hsp104 ATPase activity. Gossypol-acetic acid complex was omitted for its similarity to Gossypol.
(TIF)

## References

[1] Dobson CM (2003) Protein folding and misfolding. Nature 426: 884–890. 14685248 10.1038/nature02261

[2] Vashist S, Cushman M, Shorter J (2010) Applying Hsp104 to protein-misfolding disorders. Biochem Cell Biol 88: 1–13. 20130674 10.1139/o09-121 PMC2933416

[3] Shorter J (2008) Hsp104: a weapon to combat diverse neurodegenerative disorders. Neurosignals 16: 63–74. 18097161 10.1159/000109760

[4] van Oosten-Hawle P, Morimoto RI (2014) Organismal proteostasis: role of cell-nonautonomous regulation and transcellular chaperone signaling. Genes Dev 28: 1533–1543. 25030693 10.1101/gad.241125.114 PMC4102760

[5] Hartl FU, Bracher A, Hayer-Hartl M (2011) Molecular chaperones in protein folding and proteostasis. Nature 475: 324–332. 21776078 10.1038/nature10317

[6] Shorter J, Lindquist S (2004) Hsp104 catalyzes formation and elimination of self-replicating Sup35 prion conformers. Science 304: 1793–1797. 15155912 10.1126/science.1098007

[7] Parsell DA, Kowal AS, Singer MA, Lindquist S (1994) Protein disaggregation mediated by heat-shock protein Hsp104. Nature 372: 475–478. 7984243 10.1038/372475a0

[8] Glover JR, Lindquist S (1998) Hsp104, Hsp70, and Hsp40: a novel chaperone system that rescues previously aggregated proteins. Cell 94: 73–82. 9674429 10.1016/s0092-8674(00)81223-4

[9] DeSantis ME, Leung EH, Sweeny EA, Jackrel ME, Cushman-Nick M, et al (2012) Operational plasticity enables Hsp104 to disaggregate diverse amyloid and nonamyloid clients. Cell 151: 778–793. 23141537 10.1016/j.cell.2012.09.038 PMC3496281

[10] Shorter J, Lindquist S (2005) Navigating the ClpB channel to solution. Nat Struct Mol Biol 12: 4–6. 15689967 10.1038/nsmb0105-4

[11] Wendler P, Shorter J, Plisson C, Cashikar AG, Lindquist S, et al (2007) Atypical AAA+ subunit packing creates an expanded cavity for disaggregation by the protein-remodeling factor Hsp104. Cell 131: 1366–1377. 18160044 10.1016/j.cell.2007.10.047 PMC2211523

[12] Weibezahn J, Tessarz P, Schlieker C, Zahn R, Maglica Z, et al (2004) Thermotolerance requires refolding of aggregated proteins by substrate translocation through the central pore of ClpB. Cell 119: 653–665. 15550247 10.1016/j.cell.2004.11.027

[13] Tessarz P, Mogk A, Bukau B (2008) Substrate threading through the central pore of the Hsp104 chaperone as a common mechanism for protein disaggregation and prion propagation. Mol Microbiol 68: 87–97. 18312264 10.1111/j.1365-2958.2008.06135.x

[14] Lum R, Niggemann M, Glover JR (2008) Peptide and protein binding in the axial channel of Hsp104. Insights into the mechanism of protein unfolding. J Biol Chem 283: 30139–30150. 18755692 10.1074/jbc.M804849200 PMC2662077

[15] Lum R, Tkach JM, Vierling E, Glover JR (2004) Evidence for an unfolding/threading mechanism for protein disaggregation by Saccharomyces cerevisiae Hsp104. J Biol Chem 279: 29139–29146. 15128736 10.1074/jbc.M403777200

[16] DeSantis ME, Shorter J (2012) The elusive middle domain of Hsp104 and ClpB: location and function. Biochim Biophys Acta 1823: 29–39. 21843558 10.1016/j.bbamcr.2011.07.014 PMC3219823

[17] Franzmann TM, Czekalla A, Walter SG (2011) Regulatory circuits of the AAA+ disaggregase Hsp104. J Biol Chem 286: 17992–18001. 21454552 10.1074/jbc.M110.216176 PMC3093873

[18] Schirmer EC, Queitsch C, Kowal AS, Parsell DA, Lindquist S (1998) The ATPase activity of Hsp104, effects of environmental conditions and mutations. J Biol Chem 273: 15546–15552. 9624144 10.1074/jbc.273.25.15546

[19] Grimminger-Marquardt V, Lashuel HA (2010) Structure and function of the molecular chaperone Hsp104 from yeast. Biopolymers 93: 252–276. 19768774 10.1002/bip.21301

[20] Duennwald ML, Echeverria A, Shorter J (2012) Small heat shock proteins potentiate amyloid dissolution by protein disaggregases from yeast and humans. PLoS Biol 10: e1001346. 22723742 10.1371/journal.pbio.1001346 PMC3378601

[21] Cashikar AG, Duennwald M, Lindquist SL (2005) A chaperone pathway in protein disaggregation. Hsp26 alters the nature of protein aggregates to facilitate reactivation by Hsp104. J Biol Chem 280: 23869–23875. 15845535 10.1074/jbc.M502854200 PMC1391974

[22] Haslbeck M, Miess A, Stromer T, Walter S, Buchner J (2005) Disassembling protein aggregates in the yeast cytosol. The cooperation of Hsp26 with Ssa1 and Hsp104. J Biol Chem 280: 23861–23868. 15843375 10.1074/jbc.M502697200

[23] Shorter J (2011) The mammalian disaggregase machinery: Hsp110 synergizes with Hsp70 and Hsp40 to catalyze protein disaggregation and reactivation in a cell-free system. PLoS One 6: e26319. 22022600 10.1371/journal.pone.0026319 PMC3194798

[24] Torrente MP, Shorter J (2013) The metazoan protein disaggregase and amyloid depolymerase system: Hsp110, Hsp70, Hsp40, and small heat shock proteins. Prion 7: 457–463. 24401655 10.4161/pri.27531 PMC4201613

[25] Doyle SM, Shorter J, Zolkiewski M, Hoskins JR, Lindquist S, et al (2007) Asymmetric deceleration of ClpB or Hsp104 ATPase activity unleashes protein-remodeling activity. Nat Struct Mol Biol 14: 114–122. 17259993 10.1038/nsmb1198 PMC1793998

[26] Sanchez Y, Lindquist SL (1990) HSP104 required for induced thermotolerance. Science 248: 1112–1115. 2188365 10.1126/science.2188365

[27] Sanchez Y, Taulien J, Borkovich KA, Lindquist S (1992) Hsp104 is required for tolerance to many forms of stress. EMBO J 11: 2357–2364. 1600951 10.1002/j.1460-2075.1992.tb05295.x PMC556703

[28] Elsworth B, Matthews K, Nie CQ, Kalanon M, Charnaud SC, et al (2014) PTEX is an essential nexus for protein export in malaria parasites. Nature 511: 587–591. 25043043 10.1038/nature13555

[29] Beck JR, Muralidharan V, Oksman A, Goldberg DE (2014) PTEX component HSP101 mediates export of diverse malaria effectors into host erythrocytes. Nature 511: 592–595. 25043010 10.1038/nature13574 PMC4130291

[30] Grimminger V, Richter K, Imhof A, Buchner J, Walter S (2004) The prion curing agent guanidinium chloride specifically inhibits ATP hydrolysis by Hsp104. J Biol Chem 279: 7378–7383. 14668331 10.1074/jbc.M312403200

[31] Zeymer C, Werbeck ND, Schlichting I, Reinstein J (2013) The molecular mechanism of Hsp100 chaperone inhibition by the prion curing agent guanidinium chloride. J Biol Chem 288: 7065–7076. 23341453 10.1074/jbc.M112.432583 PMC3591616

[32] Chang L, Bertelsen EB, Wisen S, Larsen EM, Zuiderweg ER, et al (2008) High-throughput screen for small molecules that modulate the ATPase activity of the molecular chaperone DnaK. Anal Biochem 372: 167–176. 17904512 10.1016/j.ab.2007.08.020

[33] Galam L, Hadden MK, Ma Z, Ye QZ, Yun BG, et al (2007) High-throughput assay for the identification of Hsp90 inhibitors based on Hsp90-dependent refolding of firefly luciferase. Bioorg Med Chem 15: 1939–1946. 17223347 10.1016/j.bmc.2007.01.004 PMC1906718

[34] Chou TF, Brown SJ, Minond D, Nordin BE, Li K, et al (2011) Reversible inhibitor of p97, DBeQ, impairs both ubiquitin-dependent and autophagic protein clearance pathways. Proc Natl Acad Sci U S A 108: 4834–4839. 21383145 10.1073/pnas.1015312108 PMC3064330

[35] Martin I, Underhaug J, Celaya G, Moro F, Teigen K, et al (2013) Screening and evaluation of small organic molecules as ClpB inhibitors and potential antimicrobials. J Med Chem 56: 7177–7189. 23961953 10.1021/jm400499k

[36] Magnaghi P, D’Alessio R, Valsasina B, Avanzi N, Rizzi S, et al (2013) Covalent and allosteric inhibitors of the ATPase VCP/p97 induce cancer cell death. Nat Chem Biol 9: 548–556. 23892893 10.1038/nchembio.1313

[37] DeSantis ME, Sweeny EA, Snead D, Leung EH, Go MS, et al (2014) Conserved distal loop residues in the Hsp104 and ClpB middle domain contact nucleotide-binding domain 2 and enable Hsp70-dependent protein disaggregation. J Biol Chem 289: 848–867. 24280225 10.1074/jbc.M113.520759 PMC3887210

[38] Sweeny EA, DeSantis ME, Shorter J (2011) Purification of hsp104, a protein disaggregase. J Vis Exp 55: e3190. 10.3791/3190 PMC3230206 21989490

[39] Hattendorf DA, Lindquist SL (2002) Cooperative kinetics of both Hsp104 ATPase domains and interdomain communication revealed by AAA sensor-1 mutants. EMBO J 21: 12–21. 11782421 10.1093/emboj/21.1.12 PMC125804

[40] Jackrel ME, DeSantis ME, Martinez BA, Castellano LM, Stewart RM, et al (2014) Potentiated Hsp104 variants antagonize diverse proteotoxic misfolding events. Cell 156: 170–182. 24439375 10.1016/j.cell.2013.11.047 PMC3909490

[41] Zolkiewski M, Kessel M, Ginsburg A, Maurizi MR (1999) Nucleotide-dependent oligomerization of ClpB from Escherichia coli. Protein Sci 8: 1899–1903. 10493591 10.1110/ps.8.9.1899 PMC2144395

[42] Zhang JH, Chung TD, Oldenburg KR (1999) A Simple Statistical Parameter for Use in Evaluation and Validation of High Throughput Screening Assays. J Biomol Screen 4: 67–73. 10838414 10.1177/108705719900400206

[43] Heallen TR, Adams HP, Furuta T, Verbrugghe KJ, Schumacher JM (2008) An Afg2/Spaf-related Cdc48-like AAA ATPase regulates the stability and activity of the C. elegans Aurora B kinase AIR-2. Dev Cell 15: 603–616. 18854144 10.1016/j.devcel.2008.08.005 PMC2582393

[44] Halbeisen RE, Gerber AP (2009) Stress-dependent coordination of transcriptome and translatome in yeast. PLoS Biol 7: e1000105. 19419242 10.1371/journal.pbio.1000105 PMC2675909

[45] Schaupp A, Marcinowski M, Grimminger V, Bosl B, Walter S (2007) Processing of proteins by the molecular chaperone Hsp104. J Mol Biol 370: 674–686. 17543332 10.1016/j.jmb.2007.04.070

[46] Baell JB, Holloway GA (2010) New substructure filters for removal of pan assay interference compounds (PAINS) from screening libraries and for their exclusion in bioassays. J Med Chem 53: 2719–2740. 20131845 10.1021/jm901137j

[47] McGovern SL, Caselli E, Grigorieff N, Shoichet BK (2002) A common mechanism underlying promiscuous inhibitors from virtual and high-throughput screening. J Med Chem 45: 1712–1722. 11931626 10.1021/jm010533y

[48] Feng BY, Shelat A, Doman TN, Guy RK, Shoichet BK (2005) High-throughput assays for promiscuous inhibitors. Nat Chem Biol 1: 146–148. 16408018 10.1038/nchembio718

[49] Feng BY, Shoichet BK (2006) A detergent-based assay for the detection of promiscuous inhibitors. Nat Protoc 1: 550–553. 17191086 10.1038/nprot.2006.77 PMC1544377

[50] Izikki M, Mercier O, Lecerf F, Guin LL, Hoang E, et al (2013) The beneficial effect of suramin on monocrotaline-induced pulmonary hypertension in rats. PLoS One 8: e77073. 24143201 10.1371/journal.pone.0077073 PMC3797142

[51] Babokhov P, Sanyaolu AO, Oyibo WA, Fagbenro-Beyioku AF, Iriemenam NC (2013) A current analysis of chemotherapy strategies for the treatment of human African trypanosomiasis. Pathog Glob Health 107: 242–252. 23916333 10.1179/2047773213Y.0000000105 PMC4001453

[52] Borges S, Doppler HR, Storz P (2014) A combination treatment with DNA methyltransferase inhibitors and suramin decreases invasiveness of breast cancer cells. Breast Cancer Res Treat 144: 79–91. 24510012 10.1007/s10549-014-2857-2 PMC3982927

[53] Naviaux RK, Zolkipli Z, Wang L, Nakayama T, Naviaux JC, et al (2013) Antipurinergic therapy corrects the autism-like features in the poly(IC) mouse model. PLoS One 8: e57380. 23516405 10.1371/journal.pone.0057380 PMC3596371

[54] Naviaux JC, Schuchbauer MA, Li K, Wang L, Risbrough VB, et al (2014) Reversal of autism-like behaviors and metabolism in adult mice with single-dose antipurinergic therapy. Transl Psychiatry 4: e400. 24937094 10.1038/tp.2014.33 PMC4080315

[55] Trevitt CR, Collinge J (2006) A systematic review of prion therapeutics in experimental models. Brain 129: 2241–2265. 16816391 10.1093/brain/awl150

[56] Nunziante M, Kehler C, Maas E, Kassack MU, Groschup M, et al (2005) Charged bipolar suramin derivatives induce aggregation of the prion protein at the cell surface and inhibit PrPSc replication. J Cell Sci 118: 4959–4973. 16219680 10.1242/jcs.02609

[57] Stein CA (1993) Suramin: a novel antineoplastic agent with multiple potential mechanisms of action. Cancer Res 53: 2239–2248. 8485709

[58] McGovern SL, Shoichet BK (2003) Kinase inhibitors: not just for kinases anymore. J Med Chem 46: 1478–1483. 12672248 10.1021/jm020427b

[59] Morgan HP, McNae IW, Nowicki MW, Zhong W, Michels PA, et al (2011) The trypanocidal drug suramin and other trypan blue mimetics are inhibitors of pyruvate kinases and bind to the adenosine site. J Biol Chem 286: 31232–31240. 21733839 10.1074/jbc.M110.212613 PMC3173065

[60] Wigle TJ, Singleton SF (2007) Directed molecular screening for RecA ATPase inhibitors. Bioorg Med Chem Lett 17: 3249–3253. 17499507 10.1016/j.bmcl.2007.04.013 PMC1933586

[61] Maccio A, Madeddu C (2013) Cisplatin: an old drug with a newfound efficacy – from mechanisms of action to cytotoxicity. Expert Opin Pharmacother 14: 1839–1857. 23876094 10.1517/14656566.2013.813934

[62] Siddik ZH (2003) Cisplatin: mode of cytotoxic action and molecular basis of resistance. Oncogene 22: 7265–7279. 14576837 10.1038/sj.onc.1206933

[63] Ivanov AI, Christodoulou J, Parkinson JA, Barnham KJ, Tucker A, et al (1998) Cisplatin binding sites on human albumin. J Biol Chem 273: 14721–14730. 9614070 10.1074/jbc.273.24.14721

[64] Jung G, Jones G, Masison DC (2002) Amino acid residue 184 of yeast Hsp104 chaperone is critical for prion-curing by guanidine, prion propagation, and thermotolerance. Proc Natl Acad Sci U S A 99: 9936–9941. 12105276 10.1073/pnas.152333299 PMC126603

[65] Ferreira PC, Ness F, Edwards SR, Cox BS, Tuite MF (2001) The elimination of the yeast [PSI+] prion by guanidine hydrochloride is the result of Hsp104 inactivation. Mol Microbiol 40: 1357–1369. 11442834 10.1046/j.1365-2958.2001.02478.x

[66] Frydman J, Erdjument-Bromage H, Tempst P, Hartl FU (1999) Co-translational domain folding as the structural basis for the rapid de novo folding of firefly luciferase. Nat Struct Biol 6: 697–705. 10404229 10.1038/10754

[67] Shorter J, Lindquist S (2006) Destruction or potentiation of different prions catalyzed by similar Hsp104 remodeling activities. Mol Cell 23: 425–438. 16885031 10.1016/j.molcel.2006.05.042 PMC1540446

[68] Balzi E, Goffeau A (1995) Yeast multidrug resistance: the PDR network. J Bioenerg Biomembr 27: 71–76. 7629054 10.1007/BF02110333

[69] Chernoff YO, Lindquist SL, Ono B, Inge-Vechtomov SG, Liebman SW (1995) Role of the chaperone protein Hsp104 in propagation of the yeast prion-like factor [psi+]. Science 268: 880–884. 7754373 10.1126/science.7754373

[70] Liu N, Zhuang S (2011) Tissue protective and anti-fibrotic actions of suramin: new uses of an old drug. Curr Clin Pharmacol 6: 137–142. 21470104 10.2174/157488411796151174

[71] Weber-Ban EU, Reid BG, Miranker AD, Horwich AL (1999) Global unfolding of a substrate protein by the Hsp100 chaperone ClpA. Nature 401: 90–93. 10485712 10.1038/43481

[72] Schirmer EC, Ware DM, Queitsch C, Kowal AS, Lindquist SL (2001) Subunit interactions influence the biochemical and biological properties of Hsp104. Proc Natl Acad Sci U S A 98: 914–919. 11158570 10.1073/pnas.031568098 PMC14684

[73] Hattendorf DA, Lindquist SL (2002) Analysis of the AAA sensor-2 motif in the C-terminal ATPase domain of Hsp104 with a site-specific fluorescent probe of nucleotide binding. Proc Natl Acad Sci U S A 99: 2732–2737. 11867765 10.1073/pnas.261693199 PMC122416

[74] Mogk A, Schlieker C, Strub C, Rist W, Weibezahn J, et al (2003) Roles of individual domains and conserved motifs of the AAA+ chaperone ClpB in oligomerization, ATP hydrolysis, and chaperone activity. J Biol Chem 278: 17615–17624. 12624113 10.1074/jbc.M209686200

[75] Parsell DA, Kowal AS, Lindquist S (1994) Saccharomyces cerevisiae Hsp104 protein. Purification and characterization of ATP-induced structural changes. J Biol Chem 269: 4480–4487. 8308017

[76] DeSantis ME, Shorter J (2012) Hsp104 drives “protein-only” positive selection of Sup35 prion strains encoding strong [PSI(+)]. Chem Biol 19: 1400–1410. 23177195 10.1016/j.chembiol.2012.09.013 PMC3508465

[77] Carroni M, Kummer E, Oguchi Y, Wendler P, Clare DK, et al (2014) Head-to-tail interactions of the coiled-coil domains regulate ClpB activity and cooperation with Hsp70 in protein disaggregation. Elife (Cambridge) 3: e02481. 10.7554/eLife.02481 PMC4023160 24843029

[78] Gilch S, Winklhofer KF, Groschup MH, Nunziante M, Lucassen R, et al (2001) Intracellular re-routing of prion protein prevents propagation of PrP(Sc) and delays onset of prion disease. EMBO J 20: 3957–3966. 11483499 10.1093/emboj/20.15.3957 PMC149175

[79] Parsell DA, Sanchez Y, Stitzel JD, Lindquist S (1991) Hsp104 is a highly conserved protein with two essential nucleotide-binding sites. Nature 353: 270–273. 1896074 10.1038/353270a0

[80] Jackrel ME, Shorter J (2014) Reversing deleterious protein aggregation with re-engineered protein disaggregases. Cell Cycle 13: 1379–1383. 24694655 10.4161/cc.28709 PMC4050135

[81] Jackrel ME, Shorter J (2014) Potentiated Hsp104 variants suppress toxicity of diverse neurodegenerative disease-linked proteins. Dis Model Mech doi:10.1242/dmm.016113. 10.1242/dmm.016113 PMC4174528 25062688

[82] Cashikar AG, Schirmer EC, Hattendorf DA, Glover JR, Ramakrishnan MS, et al (2002) Defining a pathway of communication from the C-terminal peptide binding domain to the N-terminal ATPase domain in a AAA protein. Mol Cell 9: 751–760. 11983167 10.1016/s1097-2765(02)00499-9

[83] Schirmer EC, Homann OR, Kowal AS, Lindquist S (2004) Dominant gain-of-function mutations in Hsp104p reveal crucial roles for the middle region. Mol Biol Cell 15: 2061–2072. 14978213 10.1091/mbc.E02-08-0502 PMC404004

[84] Kummer E, Oguchi Y, Seyffer F, Bukau B, Mogk A (2013) Mechanism of Hsp104/ClpB inhibition by prion curing Guanidinium hydrochloride. FEBS Lett 587: 810–817. 23416293 10.1016/j.febslet.2013.02.011

