# Supplementary figures and images for: Suramin Inhibits Hsp104 ATPase and Disaggregase Activity
Source: PLoS One. 2014 Oct 9;9(10):e110115. doi: 10.1371/journal.pone.0110115 (PMC4192545; doi:10.1371/journal.pone.0110115)

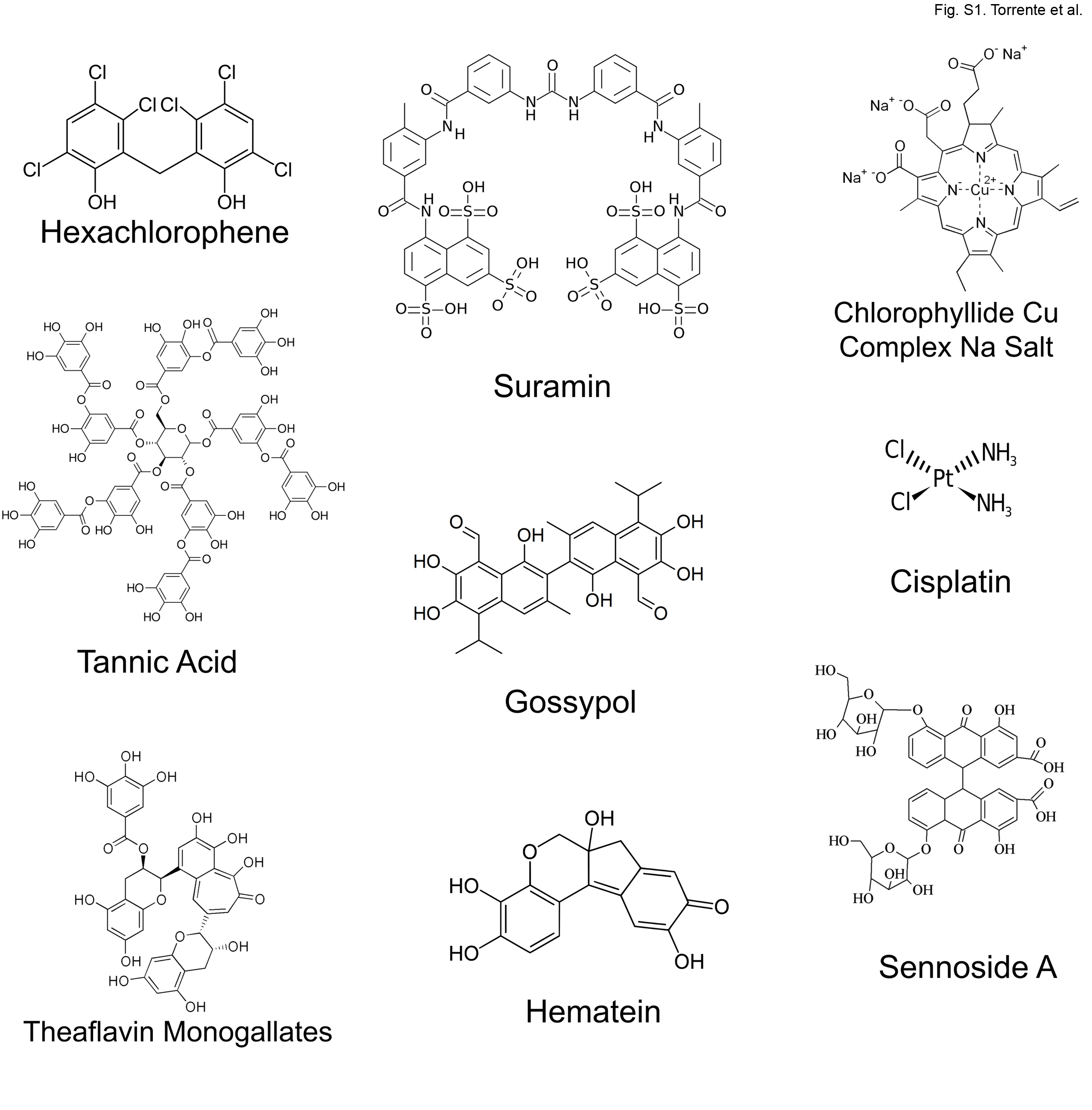

Supplement: Figure S1 — Small Molecules that Inhibit Hsp104 ATPase Activity. Chemical structures and common names are shown for nine molecules found to inhibit Hsp104 ATPase activity. Gossypol-acetic acid complex was omitted for its similarity to Gossypol. (TIF) [file pone.0110115.s001.tif]
